# Healthcare professionals’ experiences of supporting pregnant women with mental illness with their smoking behaviors: a qualitative study

**DOI:** 10.1093/ntr/ntag037

**Published:** 2026-02-19

**Authors:** Ellie Jones, Amelia Casey, Christine MacArthur, Tim Coleman, Jelena Jankovic, Beck Taylor

**Affiliations:** Department of Applied Health Sciences, School of Applied Health Sciences, College of Medicine and Health, University of Birmingham, Edgbaston, Birmingham B15 2TT, United Kingdom; Department of Applied Health Sciences, School of Applied Health Sciences, College of Medicine and Health, University of Birmingham, Edgbaston, Birmingham B15 2TT, United Kingdom; Department of Applied Health Sciences, School of Applied Health Sciences, College of Medicine and Health, University of Birmingham, Edgbaston, Birmingham B15 2TT, United Kingdom; Centre for Academic Primary Care, University of Nottingham, Nottingham NG7 2RD, United Kingdom; Birmingham and Solihull Mental Health Foundation Trust, Perinatal Mental Health Service, Birmingham B15 3RB, United Kingdom; Warwick Medical School, University of Warwick, Coventry CV4 7AL, United Kingdom

**Keywords:** tobacco control, pregnancy—treatment and intervention, mental health

## Abstract

**Introduction:**

Pregnant women who use specialist perinatal mental health services are more likely to be smokers at the time of giving birth than those without mental illness. This study explored health professionals’ experiences of providing smoking cessation support to pregnant women with complex and severe mental illness.

**Methods:**

Online video semi-structured interviews were conducted in England, October 2023-2024, with 20 National Health Service healthcare professionals whose role included discussing smoking cessation with pregnant women with mental illness. Using job descriptions, participants were categorized “signposters” (*n* = 11) or “stop smoking practitioners” (*n* = 9). Interview topic guides and data analysis were guided by the Theoretical Domains Framework.

**Results:**

Reported barriers to effective provision of smoking cessation support included prioritizing mental health over smoking cessation and being cautious about communicating harms of smoking in case it caused women’s mental health to deteriorate. Health professionals also lacked confidence that pregnant women with mental illness could quit. Lacking knowledge about the relationship between mental health and smoking was a barrier, specifically for many “signposters.” Facilitators included having capacity for health professionals to be responsive to women’s needs, offering flexibility, and effective integration of smoking cessation services within maternity and mental health care settings.

**Conclusions:**

Barriers to effective smoking cessation support included prioritizing mental illness over smoking cessation advice and a pessimistic view that pregnant women with mental illness would struggle to quit smoking. Facilitators included full integration of services and adapting to women’s needs by offering flexible timing and delivery of smoking cessation support.

**Implications:**

This study shows multiple areas which may be impacting on the effectiveness of smoking cessation support available to pregnant women with mental illness. Future research could address some of the barriers identified in the study including prioritizing mental health over smoking cessation, addressing health professionals’ beliefs that pregnant women with mental illness are unlikely to quit smoking, training on communicating harms of smoking and improving knowledge on effect of smoking on mental health. Future research could also focus on sharing good practice including appointment flexibility and full integration of smoking cessation services within maternity and perinatal mental health services.

## Background

Smoking is the leading preventable cause of fetal and childhood morbidity and mortality in the United Kingdom.^1,2^ Pregnant women who smoke are twice as likely to experience a stillbirth, up to a third more likely to have a miscarriage, and babies born to smokers are three times more likely to suffer from sudden infant death syndrome.^3–5^ Smoking during pregnancy also increases the risk of low birthweight, children developing respiratory conditions, attention and hyperactivity disorders, learning disabilities, ear, nose, and throat problems, obesity, diabetes, and becoming smokers themselves. In addition, considerable National Health Service (NHS) costs estimated to be between £8.1 and £64 million for treating maternal problems^6^ and between £12 and £23.5 million for treating infants.^7^

Rates of smoking in pregnancy are higher in young women and socially disadvantaged groups^8^ and women with mental illness are disproportionately affected. Although international data suggest that pregnant smokers with mental illness are more likely than those without to be offered cessation counseling,^9^ they remain significantly more likely to continue smoking in pregnancy, with studies from the United States of America (USA) reporting prevalence rates of 27%-33% among women with depression or anxiety compared to 10%-13% without,^10,11^ and almost half of pregnant smokers meeting criteria for at least one mental disorder.^12^ Consistently lower quit rates have also been observed among pregnant women with mental illness (48% compared to 62%).^13^ United Kingdom qualitative evidence further indicates that women with mental illness are just as motivated as those without to quit, suggesting that additional barriers beyond motivation and support contribute to the persistence of smoking.^14^

This mirrors trends in the general population, where smoking prevalence is higher among people with mental illness (26.8% compared to 14.5% [2018/2019] with no mental health illness).^15,16^ People with severe mental illness (SMI), such as schizophrenia and bipolar disorder, are less likely to engage with smoking cessation services^17^ more likely to smoke heavily, extracting more nicotine per cigarette, and are less likely to quit than those without mental illness, despite the same levels of motivation.

Health professionals play a crucial role in supporting pregnant women to quit smoking, with evidence that women are most successful when receiving professional support from stop smoking services,^18^ which may include the use of nicotine replacement therapy.^19,20^ In the UK, various policies and guidelines define how health professionals provide care to pregnant smokers. The Saving Babies’ Lives Care Bundle version 3 (SBLCB v3),^21^ outlines that all relevant maternity staff should receive training to use carbon monoxide monitors and how to have a brief and meaningful conversation with women about smoking. Very brief advice should include asking about whether the woman smokes, giving advice on the harms of smoking and the most effective way of stopping, and referring to stop smoking services. For stop smoking practitioners (SSPs), the National Centre for Smoking Cessation Training (NCSCT) provides training and guidance on what and how women should receive stop smoking support. There is also general guidance on how to support people with their smoking behaviors.^22^

There is a persistent high prevalence of smoking among pregnant women who are most adversely affected by mental illness, and evidence on smoking cessation interventions for non-pregnant people with SMI suggests that a bespoke approach to smoking cessation may also be beneficial for this group of women.^23^ Currently, all health professionals who provide care and treatment for women with complex and SMI within specialist perinatal mental health services are expected to encourage smoking cessation, and yet there is no guidance on how best to do this. The lack of guidance probably reflects that there is little relevant research; for example, we searched but found no literature describing health professionals’ experiences of supporting pregnant women with mental illness in smoking cessation. Hence, in this study, we investigate barriers and facilitators to health professionals offering stop smoking advice/support to pregnant women with mental illness. Our focus is on professionals who support women with complex and SMI, such as those cared for by secondary care specialist perinatal mental health services.

## Methods

### Theoretical underpinnings

The Theoretical Domains Framework (TDF) is a pragmatic framework that synthesizes constructs from multiple behavior change theories into 14 domains (eg, knowledge, social influences, goals) to support the systematic identification of influences on behavior (Atkins et al., 2017). These domains can be mapped to the COM-B model, which proposes that behavior (B) results from the interaction of capability (C), opportunity (O), and motivation (M), providing an overarching structure for understanding behaviors and informing intervention design^24^ (Supplementary Material 2). Such frameworks have been used to understand both individual and organizational barriers and facilitators to smoking cessation in pregnancy^25–27^ and have provided a foundation for smoking behavior change interventions.^28–30^ In this study, the TDF was applied to explore behaviors relating to health professionals’ provision of smoking cessation support, with mapping to COM-B to situate findings within a broader framework for behavior change. This addressed the first stage of the intervention design process to define the problem in behavioral terms.^31^

### Participants and recruitment

We sought to interview health professionals who care for pregnant women with complex and SMI and who are eligible to use specialist perinatal mental health services. Health professionals were recruited from four NHS sites across England (two mental health trusts and two providing maternity care), through social media and via a mailshot from NCSCT. Maximum variation sampling was used to purposively include a diverse range of health professional job types, as the views and experiences were likely to vary depending on primary job role. Health professionals interested in taking part provided their contact details and job type, and the study research team contacted them by phone call and/or email to provide them with a study information sheet. Participants provided written or audio-recorded verbal consent prior to being interviewed. As interviews progressed, particular attention was given to specifically recruiting particular professions in line with the maximum variation sampling strategy.

Ethical approval was obtained from Chelsea and Westminster NHS Ethics Committee (23/LO/0360).

### Procedures

Semi-structured interviews were conducted and audio-recorded via Microsoft Teams by two female researchers experienced in qualitative research. Following piloting, interviews were guided by a topic guide based on the TDF and designed to explore various health professional roles in providing smoking cessation information to pregnant women with mental illness (Supplementary Material 1). People with lived experience were involved with creation and testing of the topic guide. E.J. and A.C. made field notes throughout data collection, allowing refining of the topic guide. As interviews progressed, further questions were added to the topic guide around integration of smoking cessation services within maternity settings, adding more open questions about job role and asking about family provision of smoking cessation support. Participants were given a £10 shopping voucher for taking part.

Sample adequacy was determined using the information power framework.^32^ Given our specific research aims, richness of data, and grouping of participants as signposters or SSP, the resulting sample was deemed to provide sufficient information to answer the research questions.

Recordings were transcribed verbatim and anonymized.

### Analysis

Data analyses occurred alongside data collection to refine the focus of data collection and allow identification of interpreted themes. E.J. and A.C. read and reread transcripts and independently coded them using Thematic Framework Analysis.^33^ This method of analysis was chosen as it provided a systematic yet flexible approach for managing, reducing, and analyzing the data, enabling both deductive and inductive theme development in applied health research.^33^ Codes were then combined into themes/subthemes and mapped to one of the 14 TDF domains.

E.J. and A.C. held regular discussions to resolve disagreements and discussed any uncertainties with the research team (T.C., B.T., and C.M.). Once the final coding framework had been agreed, the coding framework was applied to all transcripts to summarize the data, ensuring any important quotes or subthemes missed or misallocated were identified.

Data were categorized by job type, informed by distinctions in National Institute for Health and Care Excellence (NICE) guidance (NICE 2021) and SBLCBv3 guidance on responsibilities for supporting pregnant women with smoking behaviors. For analytic purposes, professionals providing brief advice, screening, and referral were labeled *“*signposters,*”* and those delivering cessation treatment and support were labeled *“*specialist stop smoking practitioners*” (SSP)*; these are not formal role titles but analytic categories used to distinguish broad functions. While specific roles differed between signposters and SSP, identifying the influences on their behavior to better understand barriers and facilitators to provision of support for pregnant smokers with mental illness using the TDF framework was likely to be insightful. It was noted whether themes/subthemes arose solely among signposters or SSP roles or both.

The final step considered each theme in the context of the whole dataset. To provide suggestions for which theoretical domains might be the target for intervention, determining the most prominent themes is conventional within TDF analysis.^34^ These were identified based on the frequency with which specific views or beliefs within the domain were expressed by participants and the strength of views or beliefs within each domain that were discussed. Computer-assisted qualitative data analysis software, NVivo, was used to facilitate data management. Participants were not provided with transcripts for approval, but were provided a lay summary of the study findings via email, and were given the opportunity to provide feedback on the overall findings. The Consolidated Criteria for Reporting Qualitative Studies guidance has been used to report the study^35^ (Supplementary Material 3).

### Reflexivity statement

EJ is a non-smoker and qualified midwife with experience in maternity care and qualitative interviewing, and her professional background informed a sensitive but distinct approach to interviews. A.C. is a non-smoker, research associate, and psychology master’s student with qualitative interview experience. Neither researcher had prior relationships with participants, and participants were not informed of E.J.’s midwifery registration.

## Results

We contacted 44 healthcare professionals who expressed interest in taking part, and 20 subsequently participated in an interview between October 2023 and 2024 (Table 1). Average duration of interview was 33 minutes. Five themes describe the most prominent barriers and facilitators to how health professionals described supporting women with mental illness with their smoking behaviors: *prioritizing mental health over smoking cessation, additional challenges for women being able to stop smoking, being responsive to women’s needs, integration of stop smoking services with maternity and mental health care*, and *knowing about smoking and mental health* (Supplementary Material 2). Themes and subthemes were relevant for both signposters and SSP, although there was variation in the strengths of these beliefs between job types, and whether a factor acted as a barrier or a facilitator could vary with these.

**Table 1:** Participant characteristics.

**Job role**	***n***
**Specialist smoking cessation advisor role**	
Stop smoking practitioners	4
Specialist public health midwives	4
Pharmacist	1
**Signposter role**	
Obstetrician	1
General practitioner	1
Psychiatric trainee	1
Clinical psychologist	1
Hospital antenatal clinic midwives/community midwife	3
Mental health nurse	2
Maternity care assistant	2
**Region:**	
East of England	1
London	3
South East	4
South West	3
Midlands	4
North West	4
North East and Yorkshire	1
**Recruited:**	
National Centre for Smoking Cessation Training mailshot	15
NHS Sites	4
Social Media	1

### Prioritizing mental health over smoking cessation

Both signposters and SSP described various views about whether smoking cessation should be prioritized for pregnant women with mental illness they supported. Their accounts suggested that it impacted on how they navigated building a therapeutic relationship and how they discussed the harms of smoking, with an overall need to avoid any deterioration of mental health.

#### Mental health vs stop smoking

Some participants felt smoking cessation was important to improve women’s mental and physical health, and therefore, mental illness was not perceived as a barrier to supporting women with their smoking behaviors, whereas others reported finding it difficult to know what to prioritize. Some participants described that improving mental health was prioritized over smoking cessation support, with some describing that an individualized approach was necessary. There was no pattern in the views of signposters relative to SSP in relation to their views about what should be prioritized.

*‘It really is about looking at the individual in front of you and actually, if somebody has a mental health diagnosis and is particularly unwell with it at that point, then maybe there are other priorities at that time’. SSP ID8*

#### Caution when counseling about harms

To build the therapeutic relationship with the woman, participants in both signposter and SSP roles reported cautiously talking to women with mental illness about the importance of stopping smoking. This was perceived to be important to support women to engage and attend subsequent appointments; however, participants who described this didn’t describe that once the relationship was built that they were able to talk more freely about smoking.

*‘When we are trying to build a therapeutic rapport with somebody who is mentally unwell you have got to be really aware that if you come across really negative in their eyes, you are not going to be able to develop that relationship to try and help them work on their mental state’ Signposter ID6*

Participants described techniques to broach the topic of harms of smoking with women with mental illness, although there were also participants across both groups who reported giving all the information about harms of smoking.

#### Concern to avoid deterioration of mental health

Participants across both groups did describe fear of making women with mental illness feel guilty when discussing smoking cessation and the harms of smoking, although this was reported much more in the SSP group. Some described concern that discussing harms of smoking and smoking cessation could cause women’s mental health to deteriorate. One participant reported witnessing a deterioration in mental health when they provided information about the risks of smoking in pregnancy. Concern to avoid deterioration of women’s mental health was clearly described as an important consideration when providing care to women.

*‘It's a very fine line between what you tell them and what, or how you tell them because like one lady was like beside herself with being told the risks of smoking in pregnancy. It just made her even more anxious’. SSP ID13*

### Additional challenges for pregnant women being able to stop smoking

Both signposters and SPPs reported multiple reasons why women with mental illness might not be able to stop smoking, possibly indicating that some perceived their efforts to promote smoking cessation were unlikely to work. Additional challenges that some participants believed women with mental illness they supported experienced were: the need to use smoking as a stress reliever, cultural norms within the family, coexisting addictions, poor mental health, and financial and housing issues. In light of the additional challenges described, participants across both groups were not very optimistic that pregnant women with mental illness would be able to quit if they wanted to. Optimism around pregnant women with mental illness being able to quit was perceived by participants to be highly dependent on the additional support available to them.

A few participants who described positivity around women’s ability to quit smoking cited their own success stories of women they have supported and how they used these to counter preconceived prejudices held by colleagues that these women won’t be able to quit.

*I would say the ones that don’t quit smoking and we don’t manage to successfully get to quit smoking, I would say a good proportion of them have underlying mental health conditions and reasons why they smoke. And often it’s because they’ve been let down from other services because we haven’t been able to catch their mental health support in time’ SSP ID4*

Participants reported discussion of additional challenges that women faced would often take priority in any consultations and links with the theme “*being responsive to women’s needs”* as many participants described discussing other issues in women’s lives, as well as addressing smoking.

### Being responsive to women’s needs

This theme describes how participants offered flexibility and made allowances for women with mental illness in supporting them with their smoking behaviors.

#### Ambivalence about abrupt quitting

Participants in signposter roles described actively encouraging women to reduce the number of cigarettes smoked. Reducing was considered a positive step toward a healthier pregnancy with better outcomes for the unborn baby.

By way of contrast, SSP sometimes described that reduction was not a good idea due to continued risks of smoking during pregnancy, and the primary goal should be to stop smoking. Other SSPs thought that reduction was a positive behavior change, but ultimately, setting a quit date was better. Some SSPs described they didn’t feel that focus on a quit date was beneficial for women, and that pressure to set a quit date made it more difficult for women. There was acceptance that for some women, it could be counterproductive to focus on setting a quit date, and for some women, only reduction is possible, so it might be better to encourage that.

*‘Sometimes people are not successful in their quit attempts, but they have managed to reduce their smoking…we should be saying, well quitting really is the safest thing. However, in the back of my mind I’m also thinking, well it’s not a more harmful choice than what they were doing before, so, is it potentially enough? Is it moving them a little further along their journey to becoming quit eventually?’ SSP ID8*

#### Offering flexibility

Participants in both groups described being flexible in when, where, and how conversations about smoking cessation took place. This was explained in more detail by the SSP, who offered flexibility in where the appointment took place (eg, at home) or length of the consultation. This flexibility was described as important for improving the therapeutic relationship and to improve service engagement.

A few SSPs also described flexibility in when smoking cessation was discussed, sometimes describing that other issues affecting women may be spoken about during the consultation, rather than smoking cessation.

*‘We would offer them options of appointment, location of appointments that would suit them...whichever is going to make it easier for that lady to engage with us’. SSP ID11*

### Integration of stop smoking services with maternity and mental health care

All participants described the importance of effective communication between maternity services, mental health services, and stop smoking services. Integration of stop smoking services within either mental health or maternity services was viewed positively as it meant that they could follow up with the woman and further support them with smoking cessation, for example, accessing nicotine replacement therapy prescriptions.

Geographical distance to stop smoking services was not always a barrier to effective multidisciplinary teamwork if effective IT systems allowed two-way communication between services. Participants who were working in NHS trusts with poor integration described that lack of capacity to share documentation between services meant it was not possible to follow up what happens once referred to stop smoking services.

A few participants reported that poor relationships between different types of health professionals and services were a barrier to supporting pregnant women to fully engage with stop smoking services. For example, one participant said that inadequate systems in place to allow communication between other health professionals meant it was not possible to find out whether a woman has engaged with stop smoking services or not.

*‘If they disclose that they smoke at booking, we do the referral, but…there’s no trail to follow…it’s all separate and we don’t actually get any notification of the outcome’ Signposter ID10*

### Knowledge of smoking and mental health

While nearly all participants described receiving either very brief advice smoking cessation training or full NCSCT training (depending on job role), some participants in signposter roles reported lacking knowledge about the effect of smoking on mental health.

*‘I don’t think I know enough, if I’m being quite honest’ Signposter ID10*

This contrasted to some SSPs who had completed additional training with NCSCT about mental health and smoking cessation and stated awareness of the relationship between quitting smoking, improvement of mental health, and the nicotine withdrawal cycle. These participants also described this additional training as helpful. Some participants felt that specific training on how to support women with smoking cessation would be helpful, in addition to some describing that specific training would also support with prescription of medication to support with smoking cessation.

## Discussion

This is the first known study exploring experiences of health professionals who provide care for pregnant women with complex and SMI who use specialist perinatal mental health services, and so are tasked with offering stop smoking support to those who smoke. Our study shows numerous barriers affecting health professionals’ capacity to provide smoking cessation support, including: prioritizing mental health over smoking cessation, additional challenges that pregnant women face, resulting in health professionals lacking confidence that they can stop smoking, and lack of knowledge about the relationship between mental health and smoking. Conversely, facilitators of effective smoking cessation support were: capacity for health professionals to be responsive to women’s needs, offering flexibility and effective integration of smoking cessation services within maternity and mental health care settings.

Using the TDF enabled a comprehensive exploration of health professionals’ behavioral influences in supporting pregnant women with mental illness with smoking cessation, highlighting barriers and facilitators that could be addressed in future intervention development research. Seeking the experiences of a wide range of health professionals across the country, with an opportunity to feedback on the findings, is another strength. While multiple recruitment methods were adopted, more participants were recruited through the NCSCT than other sources, and therefore, those who participated may have been more inherently interested in smoking cessation than those who had not registered or received NCSCT training. Further, we did not specify particular job roles and, therefore, health professionals who did not perceive smoking cessation to be their role (which could be a barrier to offering it) may not have opted to participate in the study.

Some health professionals described prioritizing women’s mental health over smoking cessation, and this is consistent with findings from a systematic review exploring health professionals’ attitudes toward smoking cessation interventions for people with mental illness in the general population.^36,37^ Prioritizing mental health was described predominantly by health professionals in a signposter role in our study and this may be particularly relevant to understanding the barriers to subsequent engagement with smoking in pregnancy services. Most participants also described lack of confidence that women with mental illness could quit owing to their poor mental health and additional challenges. This is consistent with other studies highlighting health professionals’ misconceptions about the ability of patients within mental health settings to quit.^36,37^ Further work addressing misconceptions about the ability of pregnant women with mental illness to quit and how to support health professionals to navigate the additional challenges these women face alongside smoking cessation support would be beneficial.

Health professionals in our study described the need to build a relationship with the woman, meaning that sometimes, talking about the harms of smoking was not prioritized. This finding is consistent with other studies exploring barriers to health professionals supporting pregnant women generally (not just those with mental illness) with smoking behaviors, which have shown that midwives prioritize a therapeutic relationship and therefore downplay the risks of smoking in pregnancy and sometimes fail to emphasize the importance of stopping smoking.^38–42^ Given that participants described a fear of making women’s mental health worse and a lack of awareness of the evidence that stopping smoking can improve mental health,^43^ further training to equip health professionals with the skills to have open discussions with women with mental illness about the harms of smoking could be beneficial.

Our study found that some health professionals were ambivalent about encouraging this group to set quit dates, currently a central tenet of stop smoking support. Efforts women made to reduce number of cigarettes smoked were mostly seen positively by health professionals, particularly the signposters group. This could be as a result of the SSP training, which focuses heavily on abrupt quitting being the only safe way to stop; however, signposters may be reflecting on the possibility that reducing is the only positive behavior change that some women can manage. For signposters, there was a recognition that reduction may be the only option for pregnant women with mental illness. Currently, the World Health Organization states there is no safe level of smoking in pregnancy^44^ and therefore current guidance specifies supporting pregnant women to quit smoking.^22^ Despite this, there is strong evidence reducing smoking for women who can’t stop is very likely to be better for maternal and infant health due to the dose-dependent associations between intensity of smoking, fetal growth^45^ birthweight^46,47^ and adverse pregnancy and neonatal outcomes.^48^ In light of this, our findings suggest a more flexible approach may be helpful for women with mental illness, and support findings from another qualitative study in England exploring pregnant women’s views of using nicotine replacement therapy to support reduction rather than quitting.^49^

In terms of facilitators of effective smoking cessation provision, offering flexibility in where and how conversations about smoking cessation are made was described as crucial, particularly for SSPs who felt this improved therapeutic relationships and overall engagement with the woman. Integration and effective communication between smoking cessation services, maternity, and perinatal mental health were also perceived to greatly facilitate smoking cessation support available for women. While other studies have reported similarly in terms of integration of smoking cessation generally within maternity settings^42^ our study highlights the potential benefit of a multidisciplinary care approach between maternity, mental health, and smoking cessation services, particularly as quitting smoking may have implications for metabolism of some psychotropic medications.^50^

## Conclusions

Using a theory-informed framework, this study identified numerous barriers to supporting women with mental illness with their smoking behaviors, including prioritizing mental health over smoking cessation advice and pessimism that pregnant women would be able to stop smoking. Facilitators included being responsive to women’s needs by being flexible about when and how smoking cessation advice is given. Pregnant women with mental illness are much more likely to smoke throughout pregnancy than those without mental illness, and addressing barriers identified is crucial to tackling long-term health inequalities.

## Supplementary Material

ntag037_Supplemental_Files

## Data Availability

Data are not available for sharing due to ethical approval limitations.
