# Supplementary material for: Healthcare professionals’ experiences of supporting pregnant women with mental illness with their smoking behaviors: a qualitative study
Source: Nicotine Tob Res. 2026 Feb 19;28(10):1666–73. doi: 10.1093/ntr/ntag037 (PMC13601961; doi:10.1093/ntr/ntag037)
Supplement: Supplementary file 1 [file ntag037_supplemental_files.zip › Supplemental_Material_1_Interview_topic_guide_v2.0250423_ntag037.docx]

Influences on smoking behaviours from the perspectives of pregnant smokers with moderate to severe mental illness and health professionals: A qualitative study using the Theoretical Domains Framework

*[The questions in the interview schedule are flexible and will be iterated as the study progresses in line with best practice]*

**Topic guide – Health professionals’ Interview Schedule**

Introduction: The general aim of this interview is to talk about your thoughts around supporting pregnant women with mental illness to stop smoking. I am here to try and improve the support available to pregnant women with mental illness who smoke so please feel free to be honest with your answers. I am also interviewing pregnant women who smoke and have mental illness to find out their views and experiences of smoking/stopping smoking. Before we start the interview, I just have a few things to check with you:

Pre interview questions:

1) Have you had access to a copy and read the PIS?
2) Have you signed a consent form (if not, go through consent form)
3) Do you have any further questions?
4) Are you happy to proceed with the interview?

Main interview

I now have some more specific questions about your thoughts around how to best to support pregnant smokers with mental illness to stop smoking. The questions are broken down into sections and it may seem like there is some overlap between the sections and what is being asked, but please just try to respond with whatever comes to mind. You can take a break or stop the interview at any time. Are you ready to start?

| **Domain** | **Theoretical constructs represented within each domain** | **Link to COM-B** | **Interview questions** |
| --- | --- | --- | --- |
| **Knowledge** (an awareness of the existence of something) | Knowledge (including knowledge of condition/scientific rationale); procedural knowledge of task environment | Capability—psychological | 1. ***What do you know about the effects of smoking on mental health****?* 2. *What do you know about VBA for smoking cessation?* |
| **Skills** (an ability or proficiency acquired through practice) | Skills; skill development; competence; ability; interpersonal skills; practice; skills assessment | Capability – physical | 3. ***In your current job role, how do you support pregnant women with mental illness with their smoking behaviours****?* 4. ***Do you know how and where to refer women to SiP services****? PROMPT guidance says should be opt-out* 5. ***Do you know how to provide information to pregnant women about smoking****? PROMPT VBA* |
| **Memory, attention and decision Processes** (the ability to retain information, focus selectively on aspects of the environment and choose between two or more alternatives) | Memory; attention; attention control; decision making; cognitive overload/tiredness | Capability – psychological | 6. ***Is smoking cessation advice something that you usually provide to pregnant women with mental illness who smoke?*** *PROMPT VBA smoking cessation* |
| **Behavioural regulation** (anything aimed at managing or changing objectively observed or measured actions) | Self-monitoring; breaking habit; action planning | Capability – psychological | 7. ***Are there systems in place for monitoring whether you have provided support/referred women to SiP services?*** |
| **Social/professional role and identity** (a coherent set of behaviours and displayed personal qualities of an individual in a social or work setting) | Professional identity; professional role; social identity; identity; professional boundaries; profession confidence; group identity; leadership; organisational commitment | Opportunity – social | 8. ***Do you feel that providing smoking cessation advice is part of your professional role?*** 9. *Is it compatible or in conflict with your professional standards or identity?* |
| **Beliefs about capabilities** (acceptance of the truth, reality, or validity about an ability, talent, or faculty that a person can put to constructive use) | Self-confidence; perceived competence; self-efficacy; perceived behavioural; beliefs; self- esteem; empowerment; professional confidence | Motivation – reflective | 10. ***How difficult/easy is it for you to provide information about smoking cessation to pregnant women with mental illness?*** |
| **Optimism** (the confidence that things will happen for the best or that desired goals will be attained) | Optimism; pessimism, unrealistic optimism; identity | Motivation – reflective | 11. ***How confident are you that pregnant women with mental illness will be able to stop smoking if they try?*** |
| **Beliefs about consequences (**acceptance of the truth, reality or validity about outcomes of a behaviour in a given situation) | Beliefs; outcome expectancies; characteristics of outcome expectancies; anticipated regret; consequents | Motivation – reflective | 12. ***What do you happens when you give women smoking cessation advice?*** 13. ***What do you think would happen if you changed how you offered pregnant women with mental illness smoking cessation support?*** |
| **Intentions** (A conscious decision to perform a behaviour or a resolve to act in a certain way) | Stability of intentions; stages of change model; transtheoretical model and stages of change | Motivation – reflective | 14. *Do you routinely give pregnant women with mental illness smoking cessation advice if they disclose to you that they smoke? PROMPT e.g. community midwives should give VBA to all women who smoke* |
| **Goals** (mental representations of outcomes or end states that an individual wants to achieve | Goals (distal/proximal); goal priority; goal/target setting; goals (autonomous/controlled); action planning; implementation intention | Motivation – reflective | 15. ***How does target setting affect how you provide smoking cessation advice? E.g. mandatory reporting?*** |
| **Reinforcement** (Increasing the probability of a response by arranging a dependant relationship, or contingency, between the response and a given stimulus) | Rewards (proximal/distal valued/not valued, probable/improbable); incentives; punishment; consequents; reinforcement; contingencies; sanctions | Motivation – automatic | 16. ***Are there any incentives to provide smoking cessation advice to pregnant women?*** *PROMPT: auditing/team praise* |
| **Emotion**  A complex reaction pattern, involving experiential, behavioural, and physiological elements, by which the individual attempts to deal with a personally significant matter or event | Fear; anxiety; affect; stress; depression; positive/negative affect; burn-out | Motivation – automatic | 17. ***How do you feel with you provide smoking cessation information to pregnant women with mental illness?*** *E.g. anger/upset/frustration/happiness/contentedness* |
| **Environmental context and resources**  Any circumstance of a person’s situation or environment that discourages or encourages the development of skills and abilities. Independence, social competence, and adaptive behaviour | Environmental stressors; resources/material resources; organisational culture/climate; salient events/critical incidents; person x environment interaction; barriers and facilitators | Opportunity - physical | 18. ***To what extent do resources facilitate or hinder your ability to provide smoking cessation information to pregnant women ?*** *PROMPT. E.g. how do you find providing VBA/ Time/motivation/rapport/prioritising mental health?* 19. *How do you feel about implementing the mandatory VBA training?* |
| Social influences  Those interpersonal processes that can cause individuals to change their thoughts, feelings, or behaviours | Social pressure; social norms; group conformity; social comparisons; group norms; social support; power; intergroup conflict; alienation; group identity; modelling | Opportunity - Social | 20. ***To what extent does what your colleagues do affect how you provide information to women/ about smoking cessation?*** *Eg. Don’t mention SC advice for women with mental illness* |

Thank you for your answers, that is all the questions I have. Do you have anything else that you would like to add? Thank you for your time and honesty.

Would you like to be informed about the progress of the study and study findings? Would you like to be invited to any events relating to the project in the future?

Organise sending of voucher for time and contribution via email/post.
