# Supplementary material for: Healthcare professionals’ experiences of supporting pregnant women with mental illness with their smoking behaviors: a qualitative study
Source: Nicotine Tob Res. 2026 Feb 19;28(10):1666–73. doi: 10.1093/ntr/ntag037 (PMC13601961; doi:10.1093/ntr/ntag037)
Supplement: Supplementary file 1 [file ntag037_supplemental_files.zip › Supplementary_material_2,_Table_2_ntag037.docx]

Supplementary material 2, Table 2. Main domains and subthemes relevant to health professionals supporting pregnant women with MSMI with their smoking behaviours with illustrative quotes.

| **Theme** | **Subtheme** | **Theoretical Domain (COM-B)** | **Illustrative quotes** | |
| --- | --- | --- | --- | --- |
|  |  |  | **Signposters** | **Stop smoking practitioners** (SSP) |
| **Prioritising mental health over smoking cessation** | **Mental health versus stop smoking** | *Goals, Professional role*  *(Reflective motivation)* | *‘It’s really important. And it might help their mental health if they give up smoking’*  *‘I think it is very important for us to encourage people to consider smoking and quitting because it is so detrimental to people’s health, but I also know working in mental health people have very little control over their situation and if the one thing they can control is the cigarettes and…for some people, they feel not smoking causes their mental health to deteriorate so, it’s a real difficult balance’ ID6*  *‘If you’re looking at it from a medical point of view, you would rather manage and solve their mental illness than you would want to stop their smoking but then further on down the line, we’ll probably have to deal with smoking-related issues…It’s really difficult. What do you treat first? ID7*    *‘I think it’s very clear that it needs to be because of the problems, the complications and the outcomes from smoking during pregnancy’ ID12* | *‘It really is about looking at the individual in front of you and actually, if somebody has a mental health diagnosis and is particularly unwell with it at that point, then maybe there are other priorities at that time’*. *SSP ID8*  *‘Conversations that I’ve had with some of my colleagues, particularly those who work in mental health, they sometimes almost suggest that it would be unethical to expect a person to quit smoking, whilst they have a mental health diagnosis’ ID8*    *‘I have a lot of different clients with different environmental and social backgrounds and sometimes stopping smoking isn’t on their priority… It really is about looking at the individual in front of you and actually, if somebody has a mental health diagnosis and is particularly unwell with it at that point, then maybe there are other priorities at that time’ list’ ID9* |
|  | **Caution when counselling about harms** | *Cognitive and interpersonal skills*  *(Psychological capability)* | *‘When we are trying to build a therapeutic rapport with somebody who is mentally unwell you have got to be really aware that if you come across really negative in their eyes, you are not going to be able to develop that relationship to try and help them work on their mental state’ Signposter ID6*  ‘I do sort of tailor what I say, but the main thing is that every woman that smokes has the same information fed to them, whether they’ve got a mental health illness or not because it is important that they all have the same information and they’re not discriminated’ Signposter ID5  *‘Building trust was always going to be tricky, but at the same time you have a role to fulfil as a health professional, to provide that information and be honest and transparent about the effects of smoking. So it is a hard balance to strike’ ID10*  *‘Sometimes I don't do it at the start of a consultation because I want to build up a bit of a rapport first’ ID18* | ‘All I’m focused about is them wanting to come back and see me again, I don’t want to make them feel bad, because they already feel bad. I want them to trust me and I want them to know that they’re in a safe place where they can talk’. SSP ID3  *‘We have to try and bear that in mind and just go for it really because it has to be said, but as I said, in a non-judgemental and supportive way’*    *‘We don’t want to give them information that might upset them. That might affect that relationship that you have’ ID11*  *‘All I’m focused about is them wanting to come back and see me again, I don’t want to make them feel bad, because they already feel bad. I want them to trust me and I want them to know that they’re in a safe place where they can talk’. ID3* |
|  | **Concern to avoid deterioration of MH** | *Beliefs about consequences, emotion (Reflective and automatic motivation)* | *‘Their mental health means that it’s an aid for anxiety, so taking that away just either makes them more mentally unwell or it’s just too much of an ask’ ID19*  *‘It's that you want the information you give them to be true and reflect the severity of what could happen. But equally you don’t want to lay it on so thickly that they feel guilty because it's not about making people feel guilty’. ID18*  I do worry that it could make them feel guilty or more worried about… Or that could lead to a deterioration in their mental health, because it might be something that they use as a coping strategy for mental health deterioration or to keep their mental health stable. ID18 | ‘I think there’s a lot of fear around mental health these days, in terms of what the potential consequences are of giving somebody some information that is potentially a bit difficult to hear, what they might do to their mental health’ SSP ID8  *‘Obviously we have to be really mindful when we’ve got a lady who’s continuing to smoke and has got anxiety’ ID4*    *‘It's a very fine line between what you tell them and what, or how you tell them because like one lady was like beside herself with being told the risks of smoking in pregnancy. It just made her even more anxious’. ID13* |
| **Additional challenges** | **-** | *Beliefs about consequences, optimism (reflective motivation)* | *‘People use smoking almost as a crutch when something difficult is happening, ‘I am going to go for a cigarette, and I will come back’ because it gives them like a time out. Yeah so, it’s a bit of a challenge’. ID6*  *‘I always feel that it must be very hard to cut down or quit if your immediate house occupants smoke, because there's that constant availability. So I do think if you can involve partners, it's better...’ ID18*    *‘Those types of patients also have complex social needs, housing, financial problems and stopping smoking is like the last thing on the list that they are prepared to cover…these are not people just smoking for fun. They are people who smoke because it’s a prop for their very difficult lives’ ID19*  *‘Not very confident, not on their own’ ID10*    *‘A whole lot of them have really tried and I must tell you the truth it isn’t really easy to quite an addiction without having the necessary support strategies in place’ ID17*  Their mental health means that it’s an aid for anxiety, so taking that away just either makes them more mentally unwell or it’s just too much of an ask’ Signposter ID19  ‘I think it very much depends on the support that they get and their understanding and the approach. So probably not very confident’. Signposter ID19 | ‘Unfortunately…people have this thing that, ‘Oh, not all mental health patients will quit from smoking.’ That’s not true because I’ve got ladies who have got mental health who have managed to quit, you know’ SSP ID14  ‘The biggest issue that I'm getting outside of most smoking is housing. It's a huge issue here. Because a lot of people live in in bedsit type situations and being concerned where they're going to go after they've had their baby. That's more of a concern to them. But they're smoking. And I get it’ SSP ID13  *‘Very frequently they will report that the smoking helps them with stress and whatever it is that is causing maybe their anxiety and low mood’. ID2*  *‘if the pregnant woman is wanting to quit and her partner is still smoking that’s even harder for her’. ID2*  *‘Although it is a very important thing, they have so many other situations, homelessness, other substance misuse, domestic violence, everything like that. They’ve got so many other things that are happening that they haven’t even thought about trying to overcome that stopping smoking yet’ ID9*  ‘I*t’s having that self-belief in themselves. Now obviously, their self-belief when they’ve got mental health issues, is probably rock bottom and they genuinely just don’t believe they can do it’. ID3*    *‘I would say the ones that don’t quit smoking and we don’t manage to successfully get to quit smoking, I would say a good proportion of them have underlying mental health conditions and reasons why they smoke. And often it’s because they’ve been let down from other services because we haven’t been able to catch their mental health support in time’ID4*    *‘I feel very confident. I’ve seen it happen, but I think that the way in which you engage women with mental health issues into the programme, has to be really well considered’ ID8* |
| **Being responsive to women’s needs** | **Ambivalence about abrupt quitting** | *Goals*  *(reflective motivation)* | ‘I think reduction is definitely a step in the right direction, if you can encourage that, that is fantastic’ Signposter ID5    *‘So if it’s a support to get you there and slowly cut down and quit altogether it will be absolutely fantastic’ ID5*    *‘It’s about saying…If you can’t stop because you don’t want to stop, maybe you could reduce’* | *‘It might be that patient reduce and reduces and reduces, but never actually quits’ ID11*    ‘*sometimes people are not successful in their quit attempts, but they have managed to reduce their smoking…we should be saying, well quitting really is the safest thing. However, in the back of my mind I’m also thinking, well it’s not a more harmful choice than what they were doing before, so, is it potentially enough? Is it moving them a little further along their journey to becoming quit eventually*?’ ID8  ‘Often [women] aren’t necessarily 100% in the right headspace to quit smoking. But we just need them to get on that step in the right direction, but to then be told, ‘quit smoking’, all of a sudden it makes something that they’ve been thinking about very real and they shut down’ SSP ID4  ‘I don’t think I’m at a point where I would confidentially recommend just reducing smoking because…the optimal choice would be to quit entirely. But personally, I feel that actually we could potentially be a little more supportive of those who are reducing, rather than saying, well that’s not good enough…Although it doesn’t necessarily reduce the risk, I think in the bigger picture, it’s still a better health behaviour than what they were previously doing’ SSP ID8  ‘We don’t support any woman to smoke during the pregnancy because obviously we want them to have a safe and healthy pregnancy’ SSP ID14 |
|  | **Offering flexibility** | *Behavioural regulation, environmental, context and resources*  *(psychological capability, physical opportunity)* | ‘*If I’ve got a really good rapport and I’ve got consent, I can send them a text message just to boost them along and that’s far better than a leaflet. I think it’s finding out ways that we can modernise support potentially’ ID7*  *‘We take more time, because it's more important for them and for their baby. We give them options and we probably follow up more’. ID16*  ‘I try to go out to the garden which they have, which they usually use as a smoking area. Try not to be annoying, but I say, ‘I can help you. You say that you want to quit smoking, and how can I help you with regards to that?’  ID16 | *‘We are flexible as well. I do say to the ladies, ‘Look and also we offer home visits as well.’ I’ve got a lady who has got agoraphobia bless her, so she can’t leave her home. So I go and visit her at home.*  *‘Some of my ladies like a WhatsApp call, some of my ladies will have a Teams call. It depends on what kind of day they’re having as well. So we offer quite varied’ ID14.*  ‘We would offer them options of appointment, location of appointments that would suit them...whichever is going to make it easier for that lady to engage with us’. SSP ID11  But if a lady has got significant mental health issues then we offer a weekly appointment regardless of what the pathway is. She’s offered an additional appointment…we are flexible as well. I do say to the ladies, ‘Look and also we offer home visits as well.’ I’ve got a lady who has got agoraphobia bless her, so she can’t leave her home. So I go and visit her at home’. SSP ID14  They’ve got so many other things that are happening that they haven’t even thought about trying to overcome that stopping smoking yet. So sometimes when I do speak to them we don’t actually even talk about stopping smoking. SSP ID9 |
| **Integration of stop smoking services with maternity and mental health care** | **-** |  | ‘*So we do work quite closely but it’s not a specific maternity services based…if they disclose that they smoke at booking, we do the referral, but like I say, [yeah] there’s no trail to follow’. ID10*  *if that person takes up the referral and supports then we probably wouldn’t have any further communication.ID12*  They will put on if they’ve not been able to get hold of the lady or if they have and they’ve sent a prescription. So I’ll go, ‘Have you been and collected your prescription?’ and there’ll be like, ‘No, the prescription wasn’t there’ and then I can chase it up for them and things like that...and also if they’re not answering their phone, it’ll say like, ‘Attempted twice and still not heard.’ Signposter ID5  ‘If they disclose that they smoke at booking, we do the referral, but…there’s no trail to follow…it’s all separate and we don’t actually get any notification of the outcome’ Signposter ID10  ‘We refer patients but I myself don't remember communicating or liaising with them on a particular patient’ Signposter ID16. | *‘Once we’ve referred to the GP, we don’t really tend to hear much back from what’s gone on, which is a bit of a shame because that would be helpful’ ID3*    *‘we’ve got the in-house service we’re all using the same notes system. So like if I see a patient this afternoon and I write on her notes, the doctor can see, ‘Right, she’s seen a smoking practitioner. This is the plan.’ Whereas sorting out to community you’re not getting that same feedback and that same consistency’. ID4*  We used to refer out to smoking cessation and it was really difficult to have that feedback between the maternity services in the hospital and the smoking cessation team’ SSP ID4 |
| **Knowledge on smoking and mental health** | **-** | *Knowledge*  *(Psychological capability)* | ‘I don’t think I know enough, if I’m being quite honest’ Signposter ID10  *[the] Public Health Midwife gives yearly updates and also talks to us about how to give that advice, but very brief advice because that’s what we would do in the first instance and then refer to the Stop Smoking Service. ID12*  *It [training] wasn't mandatory. But yeah, optional, if you wanted to provide that service and advice to patients. ID15* | ‘People who’ve got mental health, they tend to smoke a lot more as well and it can be harder for them to quit. But if you can give them the right tools to quit with and the right support it does improve their mental health significantly’. SSP ID14  ‘The NCSCT that we do our training on, it’s a specialist mental health, but there’s a mental health module on there that I’ve done as well which is quite good’. SSP ID8  ‘But if we’re better educated about it and more prepared then we’d be able to tailor that approach to those women better I think’ SC ID11  *‘The actual science behind it and quitting smoking can improve your mental health’. ID8*  *‘I also know that from the bit of training that we did about mental health medication and quitting and smoking and nicotine is that actually nicotine can actually stop medication working as effectively. So if someone quits smoking then their mental health medication will work more effectively and likely need dosage changes…if we’re better educated about it and more prepared then we’d be able to tailor that approach to those women better I think’. ID11* |
